# Integrated View on the Role of Vitamin D Actions on Bone and Growth Plate Homeostasis

**DOI:** 10.1002/jbm4.10577

**Published:** 2021-11-18

**Authors:** Lieve Verlinden, Geert Carmeliet

**Affiliations:** ^1^ Clinical and Experimental Endocrinology, Department of Chronic Diseases and Metabolism KU Leuven Leuven Belgium

**Keywords:** GENETIC ANIMAL MODELS, ANIMAL MODELS, PTH/VIT D/FGF23, CELL/TISSUE SIGNALING, ENDOCRINE PATHWAYS, CELLS OF BONE, CHONDROCYTE AND CARTILAGE BIOLOGY, DISORDERS OF CALCIUM/PHOSPHATE METABOLISM

## Abstract

1,25(OH)_2_D_3_, the biologically active form of vitamin D_3_, is a major regulator of mineral and bone homeostasis and exerts its actions through binding to the vitamin D receptor (VDR), a ligand‐activated transcription factor that can directly modulate gene expression in vitamin D‐target tissues such as the intestine, kidney, and bone. Inactivating VDR mutations or vitamin D deficiency during development results in rickets, hypocalcemia, secondary hyperparathyroidism, and hypophosphatemia, pointing to the critical role of 1,25(OH)_2_D_3_‐induced signaling in the maintenance of mineral homeostasis and skeletal health. 1,25(OH)_2_D_3_ is a potent stimulator of VDR‐mediated intestinal calcium absorption, thus increasing the availability of calcium required for proper bone mineralization. However, when intestinal calcium absorption is impaired, renal calcium reabsorption is increased and calcium is mobilized from the bone to preserve normocalcemia. Multiple cell types within bone express the VDR, thereby allowing 1,25(OH)_2_D_3_ to directly affect bone homeostasis. In this review, we will discuss different transgenic mouse models with either *Vdr* deletion or overexpression in chondrocytes, osteoblasts, osteocytes, or osteoclasts to delineate the direct effects of 1,25(OH)_2_D_3_ on bone homeostasis. We will address the bone cell type–specific effects of 1,25(OH)_2_D_3_ in conditions of a positive calcium balance, where the amount of (re)absorbed calcium equals or exceeds fecal and renal calcium losses, as well as during a negative calcium balance, due to selective *Vdr* knockdown in the intestine or triggered by a low calcium diet. © 2021 The Authors. *JBMR Plus* published by Wiley Periodicals LLC on behalf of American Society for Bone and Mineral Research.

## Endocrine Loops Regulate Mineral and Bone Homeostasis

The hormonally active form of vitamin D_3_, 1,25(OH)_2_D_3_, is an important mediator of mineral and bone homeostasis. 1,25(OH)_2_D_3_, primarily produced in the kidneys, exerts its effects through the vitamin D receptor (VDR), which is most abundantly expressed in tissues involved in the maintenance of mineral and bone homeostasis, such as the parathyroid glands, intestine, kidney, and bone.^(^ ^1^ ^)^ Mutations in the VDR gene that result in a defective receptor lead to the development of vitamin D‐dependent rickets type 2, an autosomal recessive disorder characterized by the early onset of rickets, hypocalcemia, secondary hyperparathyroidism, and hypophosphatemia.^(^ ^2^ ^)^

When serum calcium concentrations drop, they elicit an elevated secretion of parathyroid hormone (PTH) by the parathyroid glands, which in turn stimulates renal synthesis of CYP27B1, the rate‐limiting enzyme that is responsible for the hydroxylation of 25(OH)D_3_ to the biologically active form 1,25(OH)_2_D_3_. In addition, PTH induces calcium mobilization from the bone, which represents a major calcium reservoir, and enhances renal calcium reabsorption. The rise in serum 1,25(OH)_2_D_3_ concentrations induces, on its turn, calcium absorption in the intestine and reabsorption in the kidney. Moreover, to preserve normocalcemia during a negative calcium balance, 1,25(OH)_2_D_3_ can mobilize calcium from the bone.^(^ ^3^ ^)^ In a first negative feedback loop regulating calcium homeostasis, elevated levels of 1,25(OH)_2_D_3_ inhibit PTH secretion by the parathyroid glands, and in a second negative feedback loop, 1,25(OH)_2_D_3_ suppresses CYP27B1 and stimulates CYP24A1 in the kidney thereby limiting its own production and enhancing its catabolism.

During the process of correcting hypocalcemia, elevated levels of PTH and 1,25(OH)_2_D_3_ induce phosphate mobilization from the bone, leading to elevated serum phosphate levels. This hyperphosphatemia is partially corrected by PTH itself by mediating internalization of the sodium/phosphate cotransporters NPT2A and NPT2C within the renal tubules, resulting in urinary phosphate wasting. In addition, increased serum phosphate levels are sensed by osteoblasts and osteocytes, leading to an elevated production of fibroblast growth factor (FGF)23. FGF23 binds to FGFR1C/αKlotho in the kidney, which transcriptionally inhibits the expression of *Npt2a* and *Npt2c* and thereby promotes phosphaturia. Furthermore, 1,25(OH)_2_D_3_ induces FGF23, and as such indirectly stimulates phosphate wasting. On the other hand, 1,25(OH)_2_D_3_ is able to transcriptionally induce *Npt2a* expression in mouse and *NPT2A/C* in human renal cell lines, suggesting a positive effect of 1,25(OH)_2_D_3_ on renal phosphate reabsorption.^(^ ^4^, ^5^ ^)^ The in vivo importance of this regulation is, however, questioned, as short‐term treatment of mice with supraphysiological doses of 1,25(OH)_2_D_3_ increases NPT2A protein levels in total renal homogenates, whereas urinary phosphate levels are not altered.^(^ ^6^ ^)^ Therefore, further research is required to establish the role of 1,25(OH)_2_D_3_ in the regulation of renal phosphate reabsorption.^(^ ^7^ ^)^ Of note, 1,25(OH)_2_D_3_ also increases the expression of NPT2B in the intestine, thereby stimulating intestinal transcellular phosphate absorption.^(^ ^8^, ^9^, ^10^ ^)^

Elevated FGF23 levels not only induce renal phosphate excretion but also affect renal vitamin D metabolism by decreasing *Cyp27b1* expression and stimulating *Cyp24a1* expression, which together reduce circulating levels of 1,25(OH)_2_D_3_. Thus, normal serum concentrations of calcium and phosphate are guaranteed by a complex interplay between 1,25(OH)_2_D_3_, PTH, and FGF23, which is controlled by multiple feedback mechanisms operating in the parathyroid glands, kidney, and bone.

## 1,25(OH) _2_D_3_ Indirectly Regulates Bone Homeostasis by Enhancing Intestinal Calcium Absorption

One of the major activities of 1,25(OH)_2_D_3_ is to stimulate intestinal calcium absorption. Adequate dietary calcium absorption indirectly contributes to bone homeostasis by ensuring sufficient calcium supply to the bone, which is crucial for bone mineralization. Intestinal calcium absorption occurs through a saturable, transcellular pathway that is energy‐dependent as well as through a non‐saturable, paracellular passive pathway. The passive transport takes place throughout the whole intestine and is dependent on the intestinal luminal calcium concentration. Although it was previously assumed that 1,25(OH)_2_D_3_ only induces transcellular calcium uptake in the duodenum, recent findings indicate a more complex situation. First, the paracellular calcium transport seems also regulated by the action of 1,25(OH)_2_D_3_, as the expression of the paracellular calcium channels *Claudin 2* and *12* is induced by 1,25(OH)_2_D_3_ and decreased in the intestine of *Vdr* null mice.^(^ ^11^ ^)^ Second, it now appears that 1,25(OH)_2_D_3_ also stimulates the transcellular process in the large intestine.^(^ ^12^ ^)^

The essential role of 1,25(OH)_2_D_3_‐mediated intestinal calcium absorption for bone and mineral homeostasis is clearly demonstrated by the phenotype of *Vdr* null mice. *Vdr* null mice are phenotypically normal at birth, but after weaning, they develop hypocalcemia, secondary hyperparathyroidism, and hypophosphatemia despite very high levels of 1,25(OH)_2_D_3_, and they become growth retarded with severe rickets.^(^ ^13^, ^14^, ^15^, ^16^ ^)^ Mice deficient in *Cyp27b1* display a similar phenotype.^(^ ^17^, ^18^ ^)^ This bone and calcium phenotype can be largely corrected by a high dietary calcium intake (especially in combination with a high lactose intake) in both knockout models, indicating the importance of 1,25(OH)_2_D_3_‐mediated intestinal calcium absorption.^(^ ^14^, ^19^, ^20^, ^21^, ^22^ ^)^ Treatment with 1,25(OH)_2_D_3_ also rescues the phenotype of *Cyp27b1* mice.^(^ ^23^, ^24^, ^25^ ^)^ In contrast to systemic *Vdr* null mice, intestine‐specific *Vdr* null mice (*Villin*‐*Vdr*‐cKO mice) are normocalcemic. In response to the reduced intestinal calcium absorption in these mice, circulating levels of PTH and 1,25(OH)_2_D_3_ are increased, which not only enhance bone resorption but also suppress bone matrix mineralization. Both processes contribute to the maintenance of normal serum calcium levels, as the increased bone resorption releases calcium from the bone, whereas the suppressed bone mineralization prevents calcium incorporation in the bone (as explained later in more detail). These findings indicate that the maintenance of normocalcemia occurs at the expense of skeletal integrity.^(^ ^26^ ^)^ Interestingly, mice with *Vdr* inactivation in the distal intestine reveal that also the large intestine significantly contributes to whole‐body calcium homeostasis.^(^ ^27^ ^)^ In agreement, transgenic expression of human *VDR* exclusively in the ileum, caecum, and colon of *Vdr* null mice prevents abnormalities in calcium and bone homeostasis, confirming the essential role of VDR‐mediated calcium absorption in the distal gastrointestinal tract.^(^ ^12^, ^28^ ^)^ Of note, *villin*‐driven overexpression of the VDR in the intestine of wild‐type mice does not improve the intestinal response to 1,25(OH)_2_D_3_ nor does it prevent the bone loss when mice are fed a low calcium diet, indicating that supraphysiological intestinal VDR levels do not provide additional benefit.^(^ ^29^ ^)^

During transcellular calcium transport, 1,25(OH)_2_D_3_ enhances the apical entry of calcium across the brush border membrane into the enterocyte by upregulating the expression of the epithelial channels transient receptor potential cation channel subfamily V (TRPV) member 6 and TRPV5.^(^ ^14^ ^)^ The intracellular calcium transfer is mainly dependent on the calbindin‐D9K protein, which is encoded by the *S100g* gene.^(^ ^30^ ^)^ The transfer of calcium from the cytoplasm to the extracellular space requires energy expenditure because of an uphill concentration gradient and an unfavorable electrochemical gradient. Both the plasma membrane calcium pump PMCA1, a low‐affinity Ca‐ATPase, and a sodium‐calcium exchanger NCX1 play important roles in this process. Although the expression of *Trpv5* and *6* as well as *S100g* is highly induced by 1,25(OH)_2_D_3_, the exact molecular mechanism of 1,25(OH)_2_D_3_‐mediated transcellular calcium transport remains elusive. Indeed, the finding that significant 1,25(OH)_2_D_3_‐inducible active intestinal calcium absorption occurs in the absence of *Trpv6* and *S100g* suggests that additional calcium transporters or channels are involved.^(^ ^31^ ^)^ Recent studies show a strong induction of *Slc30a10*, which encodes a manganese efflux transporter, by 1,25(OH)_2_D_3_ both in the proximal and distal intestine, possibly suggesting an interrelationship between vitamin D signaling and manganese efflux.^(^ ^12^, ^32^, ^33^ ^)^

Taken together, 1,25(OH)_2_D_3_‐regulated intestinal calcium absorption is crucial for mineral and bone homeostasis, although the molecular mechanisms are still incompletely characterized.

## Renal Synthesis of 1,25(OH) _2_D_3_ Contributes to Renal Calcium and Phosphate Handling

Calcium reabsorption in the kidney is an important contributor to whole‐body mineral handling and is suggested to become more dominant when intestinal calcium absorption is suboptimal or when bone turnover is low.^(^ ^34^, ^35^ ^)^ Most of the filtered calcium (98% to 99%) is reabsorbed in the kidney via paracellular and transcellular transport pathways. Calcium reabsorption occurs mainly in the proximal convoluted tubules (PCTs) (60% to 70%) and in the thick ascending loop of Henle (TAL) (20%) through a passive, paracellular transport that depends on electrochemical gradients. However, fine‐tuning of calcium reabsorption occurs in the distal convoluted tubules (DCTs). Here, active transcellular calcium transport takes place in a three‐step pathway, comparable to the process in the intestine. Apical entry is mediated by TRPV5, whereas intracellular transport to the basolateral side occurs via the calbindin proteins, S100g and CALB1. Basolateral exit occurs through the calcium‐ATPase PMCA4 and the sodium calcium exchanger NCX1.^(^ ^34^, ^36^, ^37^ ^)^ Next to PTH and FGF23, 1,25(OH)_2_D_3_ also regulates the active uptake of calcium in the DCT.^(^ ^3^ ^)^ The role of VDR in 1,25(OH)_2_D‐induced calcium reabsorption is illustrated by the reduced urinary calcium excretion in mice with intestinal‐specific *Vdr* deletion^(^ ^26^ ^)^ and the elevated urinary calcium excretion in *Vdr* null mice with transgenic expression of low levels of *Vdr* exclusively in the gut.^(^ ^35^ ^)^ Moreover, the crucial role of 1,25(OH)_2_D‐regulated renal calcium reabsorption is demonstrated by persistent hypercalciuria and reduced bone mass in *Trpv5*‐deficient mice.^(^ ^38^ ^)^

Vitamin D signaling also affects renal phosphate handling, although mainly indirectly. Upon a normal dietary phosphate intake, approximately 80% of the filtered phosphate is reabsorbed, mostly via sodium‐dependent cotransport in the PCTs. Type I (NPT1) as well as type 2 (NPT2a, NPT2c) and type 3 (PIT1, PIT2) sodium/phosphate cotransporters are present in the apical membrane of the PCTs, whereas the transport mechanism at the basolateral side remains to be identified.^(^ ^39^, ^40^ ^)^ PTH and FGF23 promote renal phosphate loss by directly decreasing the abundance of NPT2A and NPT2C. 1,25(OH)_2_D_3_ influences phosphate homeostasis indirectly by stimulating FGF23 synthesis by the osteocytes and by inhibiting PTH production in the parathyroid glands.^(^ ^3^ ^)^ Direct regulation of NPT2A and NPT2C by 1,25(OH)_2_D_3_ is demonstrated in cultured renal epithelial cells, but its in vivo significance is still undefined.^(^ ^4^, ^5^ ^)^

Thus, 1,25(OH)_2_D_3_ signaling is important for fine‐tuning renal calcium reabsorption by regulating its active transport.

## Direct Effects of 1,25(OH) _2_D_3_ on Bone Homeostasis

**Fig 1:**
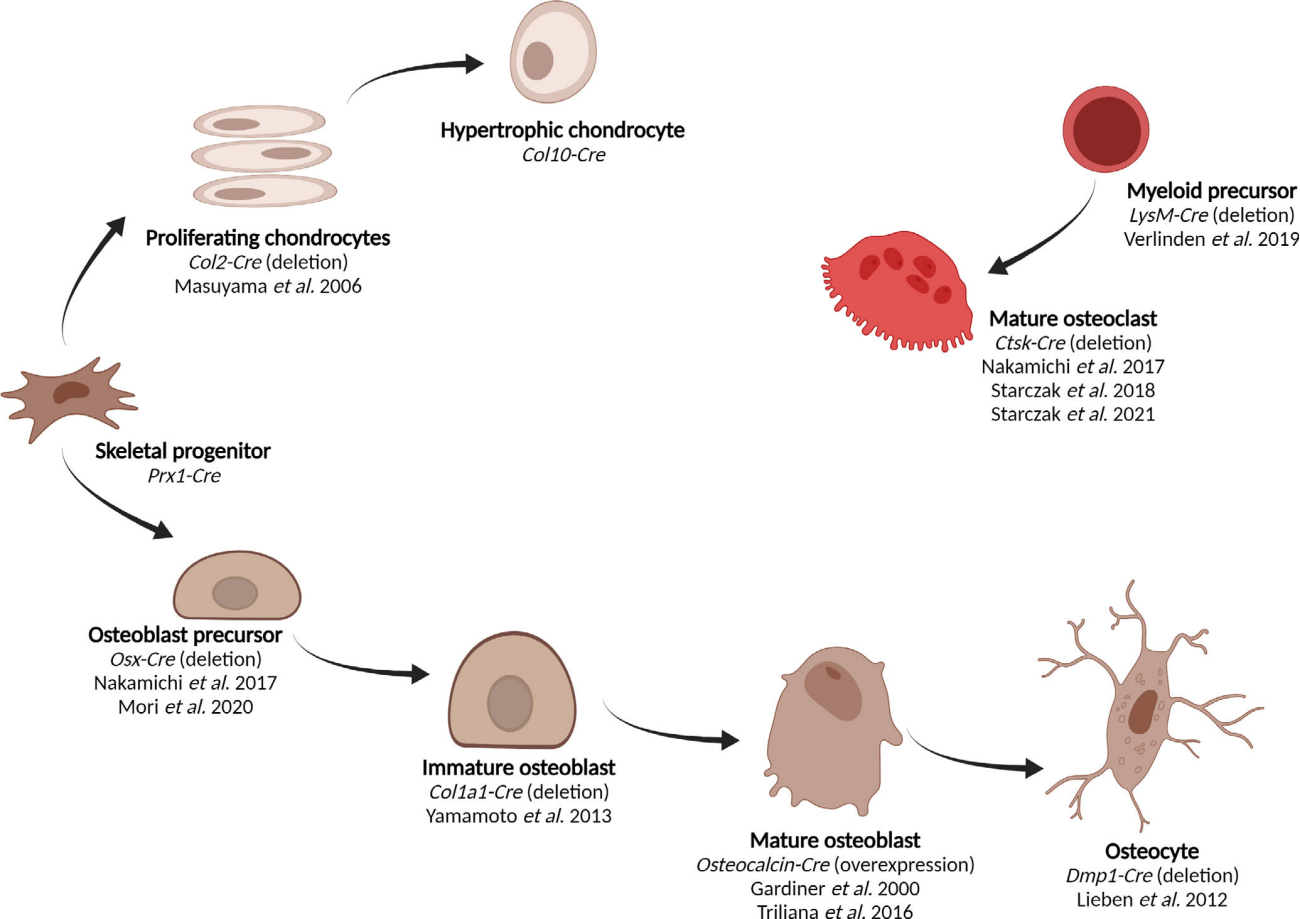
Overview of skeletal cell types in which *Vdr* expression is targeted. (Figure created with BioRender.com.)

As already outlined above, calcium malabsorption in *Vdr* null mice causes hypocalcemia after weaning and leads to the induction of hyperparathyroidism and hypophosphatemia.^(^ ^13^, ^14^, ^15^ ^)^ The deficit in calcium supply hampers bone mineralization and ultimately gives rise to the development of hyperosteoidosis and osteomalacia.^(^ ^13^, ^14^, ^15^ ^)^ Moreover, rickets will develop, characterized by an enlargement and disorganization of the zone of hypertrophic chondrocytes, which leads to a progressive widening of the epiphyseal growth plates. When calcium stores in *Vdr* null mice are replenished by feeding them a diet rich in calcium and lactose, their bone and mineral phenotype is fully restored, which emphasizes the indirect importance of 1,25(OH)_2_D_3_ for bone health by ensuring sufficient calcium (re)absorption.^(^ ^13^, ^14^, ^15^ ^)^ Nevertheless, 1,25(OH)_2_D_3_ may also directly affect bone homeostasis as chondrocytes, osteoblasts, osteocytes, and osteoclasts express the VDR. Because VDR is most abundantly expressed in osteoblasts and osteocytes, these cells are considered to be the main mediators of 1,25(OH)_2_D_3_ signaling in bone. By analysis of different transgenic mouse models with either *Vdr* deletion or overexpression in specific bone cell types, we try to delineate the direct effects of 1,25(OH)_2_D_3_ on bone homeostasis (Fig. 1). {FIG1} We will discuss the bone cell type–specific effects of VDR signaling in conditions of a positive calcium balance, where the amount of (re)absorbed calcium equals or exceeds the amount of excreted calcium (fecal or urinary loss) as well as during a negative calcium balance, resulting from insufficient calcium absorption.

### *Vdr* signaling in chondrocytes is not crucial for growth plate development but transiently regulates bone development and phosphate homeostasis

Chondrocytes develop from mesenchymal progenitor cells that have multilineage potential. These progenitors give rise to hematopoietic‐supporting bone marrow stromal cells, chondrocytes, osteoblasts, and adipocytes through highly controlled processes.^(^ ^41^ ^)^ Long bones develop through endochondral ossification, and chondrocytes in the growth plate are responsible for their lengthening, not only by proliferating but also by volume expansion during their differentiation to hypertrophic chondrocytes. During this differentiation, chondrocytes express stage‐specific extracellular matrix (ECM) proteins including different types of collagens. Promoters of these genes can therefore be used to enable chondrocyte‐specific expression of transgenes.^(^ ^42^ ^)^ As such, the *Col2a1* promoter targets resting and proliferating chondrocytes, whereas the *Col10a1* promoter marks more differentiated hypertrophic chondrocytes.

As outlined above, *Vdr* null mice develop a rachitic bone phenotype characterized by an expansion of the hypertrophic chondrocyte layers. The finding that this rachitic bone phenotype can be prevented by normalization of calcium and phosphate homeostasis indicates that the VDR is not required for normal growth plate maturation.^(^ ^43^ ^)^ Growth plate expansion in *Vdr* null mice originates from a defect in programmed cell death within the hypertrophic chondrocyte layer, as a consequence of the low serum phosphate levels.^(^ ^44^ ^)^ To investigate whether VDR in chondrocytes has a role in bone development, the consequences of *Vdr* inactivation by *Col2*‐Cre‐driven excision was investigated in *Col2*‐*Vdr*‐cKO mice.^(^ ^45^ ^)^ As expected, chondrocyte maturation occurs normally in *Col2*‐*Vdr*‐cKO mice. Yet, trabecular bone mass is increased in juvenile *Col2*‐*Vdr*‐cKO mice and is accompanied by a reduction in blood vessels and osteoclasts at the border between the growth plate and trabecular bone. This decreased osteoclast number originates from the reduced capacity of *Col2*‐*Vdr*‐cKO‐derived chondrocytes to produce receptor activator of NF‐κB ligand (RANKL) and stimulate osteoclastogenesis. In addition, serum FGF23 levels are lower in *Col2*‐*Vdr*‐cKO mice, although chondrocytes hardly express FGF23. Coculture experiments show that VDR signaling in chondrocytes indirectly regulates FGF23 secretion by osteoblasts. This reduced FGF23 level in juvenile *Col2*‐*Vdr*‐cKO mice has two consequences: first, renal *Cyp27b1* expression is increased, resulting in higher circulating 1,25(OH)_2_D_3_ levels; and second, renal NPT2A expression is enhanced, which leads to elevated serum phosphate levels. Of note, this phenotype disappears in adult mice when the contribution of VDR signaling in growth plate chondrocytes becomes less important compared with osteoblasts. In summary, these data demonstrate that VDR signaling in chondrocytes affects bone mass, by secreting RANKL, thereby inducing osteoclast formation, and it regulates phosphate homeostasis by participating in the endocrine loop between FGF23 and 1,25(OH)_2_D_3._^(^ ^45^ ^)^

### Inactivation of *Vdr* at different stages during osteoblast differentiation points to a minor role of osteoblastic *Vdr* in bone homeostasis

Osteoblasts derive from the same skeletal progenitors as chondrocytes, and their differentiation is mediated by multiple transcription factors, including Runx2 and Osterix (Osx).^(^ ^46^ ^)^ Cells in the osteogenic lineage differentiate from osteoblast precursors, to immature osteoblasts, mature osteoblasts, and finally to osteocytes. For each of these maturation stages, stage‐specific promoters can be used to silence target gene expression. The *Col1a1* promoter, which encodes the ECM protein type 1 collagen α1, is often used to target immature osteoblasts, whereas the *osteocalcin* promoter is commonly used to modify gene expression in mature osteoblasts. Terminally differentiated osteocytes, which are embedded in the bone matrix and are involved in the coordination between bone formation and resorption, can be targeted with the dentin matrix protein 1 (*Dmp1*)‐promoter.^(^ ^47^ ^)^

*Vdr* expression has been specifically deleted or overexpressed at different stages of osteoblast maturation^(^ ^26^, ^48^, ^49^, ^50^, ^51^, ^52^ ^)^ (Fig. 1). In osteoblast precursors, *Vdr* expression is successfully (90%) deleted by *Osx*‐driven recombination (*Osx*‐*Vdr*‐cKO mice), which does not alter bone mass, resorption, or formation, nor serum calcium, phosphate, 1,25(OH)_2_D_3_, or PTH levels.^(^ ^51^ ^)^ Only FGF23 levels are slightly but significantly decreased.^(^ ^51^ ^)^ Interestingly, treatment with the vitamin D analog eldecalcitol has a different effect in *Osx*‐*Vdr*‐cKO mice compared with wild‐type mice. First, eldecalcitol induces bone mass in wild‐type mice by inhibiting bone resorption, which is not observed in *Osx*‐*Vdr*‐cKO mice,^(51^ ^)^ and second, it enhances FGF23 levels in wild‐type mice but much less in *Osx*‐*Vdr*‐cKO mice, indicating that eldecalcitol‐mediated VDR signaling in osteoblast‐lineage cells suppresses bone resorption and regulates FGF23 expression.^(^ ^51^ ^)^

**Fig 2:**
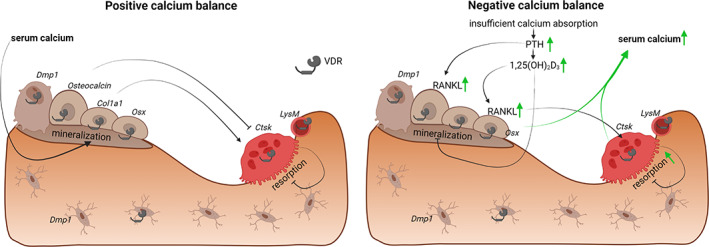
During a positive calcium balance (left panel), normal serum calcium concentrations allow proper bone mineralization. Transgenic models with *Vdr* inactivation in osteoprogenitors (*Osx*‐Cre^(^ ^51^ ^)^) or in mature osteoblasts and osteocytes (*Dmp1*‐Cre^(^ ^26^ ^)^) display normal mineral and bone homeostasis. *Vdr* knockdown in immature osteoblasts (*Col1a1*‐Cre^(^ ^49^ ^)^) induces bone mass, whereas *Vdr* overexpression in mature osteoblasts (*Osteocalcin*‐Cre^(^ ^48^, ^50^ ^)^) also leads to an elevated bone mass. *Vdr* inactivation in osteoclast precursors (*LysM*‐Cre^(^ ^57^ ^)^) does not affect bone homeostasis, whereas *Vdr*‐silencing in mature osteoclasts (*Ctsk*‐Cre) has either no effect^(^ ^51^ ^)^ or leads to a slight reduction in bone mass.^(^ ^58^ ^)^ During a negative calcium balance (right panel), insufficient intestinal calcium absorption leads to increased PTH and 1,25(OH)_2_D_3_ levels, which stimulate RANKL expression by osteoblasts and enhance bone resorption.^(^ ^52^ ^)^ Moreover, 1,25(OH)_2_D_3_ inhibits bone mineralization by directly inducing the expression of mineralization inhibitors.^(^ ^26^ ^)^ Elevated resorption and decreased mineralization both contribute to the maintenance of normocalcemia. Mice with selective *Vdr* knockdown in mature osteoclasts (*Ctsk*‐Cre^(^ ^59^ ^)^) experience a greater bone loss during a negative calcium balance. (Figure created with BioRender.com.)

Moreover, administration of high doses of 1,25(OH)_2_D_3_ to *Osx*‐*Vdr*‐cKO mice fails to increase *Rankl* expression in bone and does not lead to elevated bone resorption^(^ ^52^ ^)^ (Fig. 2). {FIG2} Together, these data indicate that VDR signaling in osteoprogenitors is not required for bone and mineral homeostasis in basal conditions, but it contributes to bone resorption when 1,25(OH)_2_D_3_ levels are increased.

Targeting *Vdr* expression in immature osteoblasts by *Col1a1*‐mediated excision (*Col1a1*‐*Vdr*‐cKO mice)^(^ ^49^ ^)^ does not affect mineral homeostasis, as evidenced by normal serum calcium, phosphate, and 1,25(OH)_2_D_3_ concentrations. In contrast to *Osx‐Vdr*‐cKO mice, *Col1a1*‐*Vdr*‐cKO mice have normal circulating levels of FGF23, possibly due to incomplete osteoblastic *Vdr* deletion (only 50%) or pointing to the importance of additional mediators that regulate serum FGF23 levels. On the other hand, adult *Col1a1*‐*Vdr*‐cKO mice show increased trabecular bone mass, accompanied by a reduced osteoclast surface due to decreased *Rankl* expression, whereas no differences in osteoblast numbers or dynamic bone parameters are present. In summary, these findings suggest that VDR signaling in immature osteoblasts has a negative effect on bone homeostasis, possibly by enhancing RANKL‐mediated osteoclastogenesis.

In contrast, *Vdr* overexpression (threefold) in mature osteoblasts under control of the *osteocalcin* promoter (*osteocalcin*‐*Vdr*‐cOE mice), evaluated on two different genetic mouse backgrounds, points to a positive effect of VDR expression in osteoblasts on bone mass.^(^ ^48^, ^50^ ^)^ Whereas serum calcium, PTH, and 1,25(OH)_2_D_3_ levels are comparable between *osteocalcin*‐*Vdr*‐cOE and wild‐type mice, cortical bone in adult *osteocalcin*‐*Vdr*‐cOE mice is wider and associated with an increased periosteal mineral apposition rate. Also, vertebral trabecular bone content is elevated in *osteocalcin*‐*Vdr*‐cOE mice, possibly due to an increased expression of *Opg*, a decoy receptor of RANKL, and subsequent decreased osteoclastic bone resorption.^(^ ^53^ ^)^

The role of VDR signaling in mature osteoblasts and osteocytes is studied by targeted *Vdr* knockdown (90%) under control of the *Dmp1* promoter (*Dmp*‐*Vdr*‐cKO mice).^(^ ^26^ ^)^ Mineral and bone homeostasis is not disturbed in *Dmp*‐*Vdr*‐cKO mice as evidenced by normal serum levels of calcium, phosphate, and 1,25(OH)_2_D_3_ and normal cortical and trabecular bone mass. Interestingly, administration of high doses 1,25(OH)_2_D_3_ increases serum calcium levels to a much lower extent in *Dmp*‐*Vdr*‐cKO mice compared with wild‐type littermates, whereas bone mass tends to be better preserved. These effects are not due to differences in osteoclastogenesis or *Rankl*/*Opg* ratios, suggesting that VDR signaling in early osteoblasts is sufficient to account for the 1,25(OH)_2_D_3_‐mediated bone resorption. On the other hand, high doses of 1,25(OH)_2_D_3_ lead to decreased mineralization of bone matrix in wild‐type mice, which is accompanied by increased expression of the mineralization inhibitors *osteopontin* and progressive ankylosis (*Ank*). These effects were not observed in *Dmp*‐*Vdr*‐cKO mice, indicating that VDR‐mediated signaling in the osteocytes impairs bone matrix mineralization in response to high circulating levels of 1,25(OH)_2_D_3_. This mechanism also contributes to the decrease in mineralized bone mass in *Vdr*‐*Villin*‐cKO mice. The *Vdr*‐*Villin*‐cKO mice represent a negative calcium balance model, with impaired intestinal calcium absorption leading to increased circulating levels of PTH and 1,25(OH)_2_D_3_. These hormonal changes likely stimulate compensatory mechanisms to preserve normocalcemia in *Vdr*‐*Villin*‐cKO mice, including increased renal calcium reabsorption, next to decreased bone calcium content, resulting from enhanced osteoclastogenesis and impaired bone matrix mineralization.^(^ ^26^ ^)^ Recently, Misof and colleagues showed that, when systemic *Vdr* null mice are given a rescue diet from weaning until the age of 4 months, bone homeostasis is normal, as previously reported. When these adult *Vdr* null mice are subsequently switched to a low calcium diet (0.5% calcium, 0.4% phosphate), they develop osteomalacia with a marked decrease in bone calcium content and an increased cortical porosity, indicative of enhanced osteoclastic bone resorption but without signs of osteocytic osteolysis.^(^ ^54^ ^)^

In summary, VDR signaling in osteogenic cells does not seem to play a major role in the control of bone homeostasis, when calcium balance is normal, although some small but discordant effects are reported. Currently, it is unclear how to reconcile the puzzling findings that both osteoblast‐specific *Vdr* deletion and *Vdr* overexpression result in a slight increase in bone mass.^(^ ^48^, ^49^, ^50^ ^)^ A possible explanation is that the effects of osteoblastic VDR signaling on bone homeostasis is dependent on the maturation stage of the osteoblasts, as suggested by in vitro data.^(^ ^55^ ^)^ Moreover, the differences in experimental settings such as gene dosage effects (deletion versus overexpression), genetic background of the mice, experimental diets, and animal age may alter VDR‐mediated signaling in bone. On the other hand, VDR signaling in osteogenic cells contributes to maintaining normocalcemia, when intestinal calcium absorption is reduced, by decreasing mineralized bone mass.

### Osteoclast‐specific VDR signaling is not a major determinant of bone homeostasis

The role of VDR signaling in osteoclasts remains controversial. Immunohistochemical analysis reveals no^(^ ^56^ ^)^ or faint^(^ ^51^ ^)^ VDR staining in osteoclasts; however, osteoclasts are shown to be responsive to 1,25(OH)_2_D_3_, as illustrated by the VDR‐mediated induction of *Cyp24a1* expression in 1,25(OH)_2_D_3_‐treated primary osteoclast cultures.^(^ ^28^, ^51^ ^)^ To investigate whether osteoclastic *Vdr* expression affects bone homeostasis, its expression was specifically deleted either in osteoclast precursors under control of the *M lysozyme* promoter (*LysM*‐*Vdr*‐cKO mice)^(^ ^57^ ^)^ or in mature osteoclasts by *Cathepsin K*‐driven Cre recombination (*Ctsk*‐*Vdr*‐cKO mice).^(^ ^51^, ^58^, ^59^ ^)^

*Vdr* inactivation in myeloid cells leads to an approximately 70% reduction of *Vdr* transcript levels in primary osteoclast cultures, derived from *LysM*‐*Vdr*‐cKO mice. Mineral and bone homeostasis are normal in young adult *LysM*‐*Vdr*‐cKO mice, as is in vitro osteoclast differentiation.^(^ ^57^ ^)^ Two research groups independently investigated the consequences of *Vdr* knockdown specifically in mature osteoclasts.^(^ ^51^, ^58^, ^59^ ^)^ Nakamichi and colleagues^(^ ^51^ ^)^ confirmed the osteoclast‐specific *Vdr* knockdown by a reduced induction of *Cyp24a1* expression in osteoclasts after stimulation with 1,25(OH)_2_D_3_, yet in vivo analysis does not reveal any alterations in bone mass of *Ctsk*‐*Vdr*‐cKO mice. However, the studies of Starczak and colleagues^(^ ^58^, ^59^ ^)^ do suggest a role of osteoclastic *Vdr* expression in the control of bone homeostasis. In their studies, *Vdr* transcript levels in whole bone homogenates of *Ctsk*‐*Vdr*‐cKO mice are reduced by 70%. Serum calcium and phosphate levels are normal in young adult *Ctsk*‐*Vdr*‐cKO mice, whereas trabecular bone mass is slightly decreased.^(^ ^58^, ^59^ ^)^ In vitro osteoclastogenesis is enhanced in *Ctsk*‐*Vdr*‐cKO‐derived splenocytes, although in vivo bone resorption parameters are normal.

The physiological consequences of osteoclastic *Vdr* knockdown are also investigated during a negative calcium balance in young adult *LysM*‐*Vdr*‐cKO^(^ ^57^ ^)^ and *Ctsk*‐*Vdr*‐cKO mice,^(^ ^59^ ^)^ by feeding a low‐calcium diet from weaning onward. Bone loss as well as osteoclast activity in *LysM*‐*Vdr*‐cKO mice induced by the low‐calcium diet are comparable to that in wild‐type littermates.^(^ ^57^ ^)^ However, trabecular bone loss is slightly more pronounced in *Ctsk*‐*Vdr*‐cKO mice on the low‐calcium diet, despite no effect on bone resorption parameters. Similar to the osteoblast‐specific *Vdr* knockdown models, differences in experimental settings of the osteoclast‐specific *Vdr* silencing (gene promoter, genetic background, animal age, experimental diet, site of bone analysis) may underlie the divergent findings in the different studies. Indeed, the *Ctsk* promoter is also expressed in other skeletal cells such as osteocytes and periosteal stem cells, which may explain the strongly reduced *Vdr* expression in *Ctsk*‐*Vdr*‐cKO bones and may account for the observed differences in bone phenotype.^(^ ^60^ ^)^

However, overall these data seem to suggest that VDR signaling in osteoclasts is not or minimally involved in bone homeostasis during a positive or a negative calcium balance.

## Conclusion

Multiple cell types within the skeleton express the VDR. High VDR expression is present in osteoblasts and osteocytes, whereas its expression is low in chondrocytes and osteoclasts.^(^ ^56^ ^)^ VDR expression in each of these different bone cell types allows 1,25(OH)_2_D_3_ to affect bone development and remodeling. During a positive calcium balance, when the amount of absorbed calcium exceeds fecal and renal calcium losses, 1,25(OH)_2_D_3_ regulates bone mass and mineralization mainly indirectly by ensuring sufficient calcium supply through intestinal and renal calcium (re)absorption.^(^ ^3^, ^61^ ^)^ At this moment, it remains unclear whether 1,25(OH)_2_D_3_ also directly affects bone homeostasis in this condition, as overexpression or deletion of *Vdr* specifically at different stages of osteoblastic differentiation yielded contradictory findings, including none, positive, and negative effects on bone resorption. Deletion of *Vdr* expression in the osteoclast lineage has little or no consequences on bone homeostasis as specific *Vdr* deletion in osteoclast precursors or in mature osteoclasts hardly affects bone resorption or alters bone mass.

During a negative calcium balance, intestinal calcium absorption does not meet the daily calcium demands required to maintain normocalcemia and to ensure proper calcium incorporation in bone. In case of intestinal calcium malabsorption, as is the case in intestine‐specific *Vdr* null mice, elevated levels of circulating PTH and 1,25(OH)_2_D_3_ stimulate production of RANKL by osteoblasts, which enhances osteoclastic bone resorption. Moreover, high 1,25(OH)_2_D_3_ levels induce the expression of mineralization inhibitors in osteoblasts and osteocytes and reduce bone matrix mineralization. The elevated bone resorption as well as the decreased mineralization contribute to the maintenance of normocalcemia by releasing calcium from bone and preventing calcium incorporation into the bone, respectively.

## Disclosures

The authors state that they have no conflicts of interest.

### PEER REVIEW

The peer review history for this article is available at https://publons.com/publon/10.1002/jbm4.10577.
